# What is Currently Understood About the Impact of Sexual Violence Activism for Higher Education Student Sexual Violence Survivors?

**DOI:** 10.1177/15248380221093691

**Published:** 2022-05-11

**Authors:** Helen Bovill, Tessa Podpadec

**Affiliations:** 1 151977 University of the West of England, Bristol, UK

**Keywords:** harm, community, labour, coalition, power, sexual violence

## Abstract

**Objective:** This systematic literature review maps the landscape of higher education and student sexual violence survivors who become involved in sexual violence activism. It was undertaken to understand what drives student sexual violence survivors to become activists, the negative and positive impacts of this activism on the students, how higher education institutions might work with sexual violence activists to foster effective prevention and response, and how activism has been negotiated by and within practice, policy and research. **Method:** A qualitative evidence synthesis methodology was used to identify research which examines drivers to and consequences of sexual violence activism for student activists. Searches across seven databases were conducted using six keywords combined in various ways, with further inclusion criteria of published in English between 2010 and 2020. Searches of grey literature were also carried out. **Results:** 28 sources met the inclusion criteria. Thematic analysis, conducted in NVivo, resulted in identification of four themes: survival from harm, community, labour in the personal made public and power between activists and institutions. **Conclusions/Recommendations/Limitations:** Inadequate institutional response was a key driver of student sexual violence activism. Activism had positive and negative impacts on the activists. Recommendations are that activists, institutions, researchers and policy makers work as coalitions to bring about enduring cultural change. Review limitations were the small number of studies in this field; additionally, they were dominated by US and UK perspectives.

## Introduction

This article is a systematic literature review mapping the landscape regarding the impact of activism on student survivors of sexual violence who take part in sexual violence activism. The aim of this review is to understand how higher education institutions might work with activists to foster lasting prevention of and effective response towards sexual violence. It is also a response to*^1^ Krause et al. (2017) who note a lack of research on campus sexual violence and student activism, particularly in the UK (*Lewis & Marine, 2018). It is important to respond to this gap in the literature as analysis of sexual violence activism can lead to development of effective prevention and response towards sexual violence on campus. Through learning from and collaborating with students who have experienced sexual violence, and who most often make up the activist community (*Linder & Myers, 2017; *Linder et al., 2016), effective change from those with experience can occur. This review will consider how, often, those who become sexual violence activists do so from a position of experience of sexual violence and because they have not gained a satisfactory response or resolution to complaints of sexual violence (for example, *Lewis et al., 2018; *Linder, 2019a; *Linder, 2019b; *Linder & Myers, 2017; *Linder, et al., 2016; *Linder et al., 2019b; *Rentschler, 2018). The review will also consider how some activists engage in activism as a form of survival (*Karasek, 2018; *Linder et al., 2019b; *Nathanson, 2014; *Taylor, 2020). Student sexual violence survivors may also turn to activism as a way to find commonality with others who have similar experiences of sexual violence and to gain some sort of understanding of this experience (*Lewis et al., 2018; *Marine & Trebisacci, 2018).

Ally activism is an important aspect of changing the landscape of sexual violence, with allyship often characterised by a member of a dominant group standing up for or by the side of those experiencing discrimination, bias or unfair treatment (Reynolds, 2011). However, the focus of this paper is on student activists with direct experience of sexual violence, and this focus upon direct experience is for various reasons. First, the literature points to most sexual violence activists coming to activism through experience (for example, *Linder & Myers, 2017; *Linder et al., 2016). Secondly, this experience can result in great losses to the activist through not just time expended on activism but also the experiences they have with disclosure, reporting and response (for example, *Bovill, et al., 2020; *Krause et al., 2017; *Linder et al., 2016; *Linder et al., 2019a; *Linder et al., 2019b; *Marine & Trebisacci, 2018).

### Activism Research

Within activism literature, some research notes that taking part in protest can have a positive effect upon activists on a wide range of measures of well-being (Boehnke & Wong, 2011; Foster, 2015; Klar & Kasser, 2009; Profitt, 2001). For example, for women sexual violence activists, engaging in collective behaviour led to increased self-confidence and an increased ability to politicise their personal experiences (Profitt, 2001). Jasko et al. (2019) reviewed a number of studies on activism including left-wing activism, feminist activism and environmental activism, finding that although negative experiences might lead to activism, taking part in collective action could result in a lessening of negative associations, stronger feelings of empowerment and a sense of positivity towards facilitating change. They also note that a level of self-sacrifice can be sustained through uniting around valued causes through collective action. Klar and Kasser (2009) conducted survey research with two sets of US college students (*N* = 341) and (*N* = 296) across a broad spectrum of activism, finding correlations between activism and positive measures of well-being. They noted that higher-risk forms of activist behaviour demonstrated less strong correlations with positive well-being measures. However, these studies are either outside of the student population and/or do not focus solely on sexual violence activism. This literature review will consider specifically the impact of sexual violence activism within the context of higher education on sexual violence survivors who become student sexual violence activists. The aim of which is to understand the role that activism might play for students in the aftermath of sexual violence and to understand how universities might work with activists to challenge a culture of sexual violence within higher education.

### Student Survivor Activism: Terms and Context

To more fully understand activism, it is useful to consider what is meant by the term, activist. *Linder and Myers (2017, p. 2) define activism as ‘organizing to transform systems of oppression for comprehensive social change’. *Marine and Trebisacci (2018, p. 649) define sexual violence activists as those who ‘work to challenge their campuses by actively opposing rape culture, changing policies and practices, and educating their peers to prevent sexual violence’. These definitions of activism demonstrate that activism may cover a spectrum of engagement from occasional attendance at demonstrations to activism which dominates the activists’ day-to-day life (*Mitra, 2015), and through this review, we found examples of this spectrum. The term activist is also more problematic when intersectionality is factored in. *Linder et al. (2019b, p. 535) highlight in their research that many of the ‘Students of Color…did not see themselves as activists…Conversely, White students…rarely hesitated to call themselves activists’. Those living in minoritised bodies had a greater tendency to view what might be termed as activism, simply as part of their life and what they had always done to survive; survival is a theme that will be explored in the discussion section of this paper.

There is also debate about the use of the terms ‘victim’ and ‘survivor’ of sexual violence which can impact upon the way that you view yourself as an activist against sexual violence. Harding (2020) considers how language can be a tool of oppression with some who have experienced sexual violence reluctant to adopt the term victim as it may imply passiveness. Equally, others may be reluctant to refer to themselves as survivors due to its connections with moving on from an experience you may not feel is, or ever will be, behind you. Although we recognise the complexities of the issue, we use the term survivor in this paper in alignment with the definition offered by Survivors Voices (n.d., p. 1):

As a shorthand for people who have experienced abuse and interpersonal trauma whilst recognising that many people with such experiences have either not heard of the term survivor or may not want to describe themselves as having been ‘abused’ or being a ‘survivor’ for many complex and valid reasons.

Finally, to put this review in context, it is important to understand the scale of the problem of sexual violence within universities, as it is the contention of this paper that understanding and working with student activists may offer a tool to reduce levels of sexual violence within the student population. Sexual violence within universities is a serious, enduring, pervasive global problem demonstrated by growing statistics, exemplified in countries such as the UK and USA which have been somewhat proactive in measuring sexual violence on campus. For example, in the UK, the National Union of Students found from a survey of 2058 women students across the UK, ‘68 percent of respondents had been subject to verbal or physical sexual harassment on campus and 14% had experienced a serious physical or sexual assault’ (Universities UK (UUK), 2016, p. 18). Revolt Sexual Assault (2018, p. 4) received 4491 responses from 153 UK institutions and found ‘70% of female students and recent graduates surveyed have experienced sexual violence’^2^. In the US, in 2018, the Association of American Universities (AAU) received 181,752 survey completions from across 33 universities, finding that overall 13% of students reported experiencing ‘non-consensual penetration, attempted penetration, sexual touching by force, or inability to consent’ (Cantor et al., 2020, p. 14). These statistics are likely representative of other countries where less research exists; for example, in a first survey of its kind of 1083 students across six universities across Spanish regions, ‘62% of the students know of or have experienced’ situations of violence against women within university institutions (Valls et al., 2016, p. 1519). The statistics are stark, such that considering activism as a tool against sexual violence is important.

This literature review maps out the existing literature on sexual violence activism on campus involving activists with experience of activism, to inform more effective prevention and response to sexual violence. Ultimately, to explore how activism by sexual violence survivors might contribute to campus cultural change in a more impactful and sustained mode than current research suggests is the case.

### Research Questions

1. What drives sexual violence survivors to become student sexual violence activists?
2. What are the key positive and negative impacts for sexual violence survivors of their student sexual violence activism?
3. What does a greater understanding of sexual violence survivors and their student sexual violence activism tell us about response by higher education institutions?

## Method

### Methodological Challenges

A systematic literature review using a qualitative evidence synthesis methodology was used for this study. Qualitative evidence synthesis follows a process of bringing together qualitative studies to understand not only ‘how people feel about an issue or a treatment but to reach an understanding of “why” they feel and behave the way they do’ (Campbell et al., 2020, p. 457, adapted from (Popay, 2005). Because this literature review is interested in the ‘how and why’ of activism rather than numbers of activists (for example), quantitative activist studies that did not have an element of qualitative analysis were part of exclusion criteria; however, in the end, no relevant quantitative sources were identified.

The terms peer review and grey literature are often used to infer a marker of quality, with peer-review literature referring to high-quality peer-reviewed records (Lawrence et al., 2014) and grey literature referring to ‘knowledge artefacts’ outside of this such as toolkits and news items (Adams et al., 2017). Alongside peer-review literature, grey literature is a key component to mapping the heterogenous corpus of literature as it can help to overcome publication bias and ‘bring the disparate voices of experience into scholarly conversation to increase its relevance and impact’ (Adams et al., 2017, p. 435). Adams et al. (2017) note that tiers between peer review and grey literature often overlap. We have included journal articles, monographs and edited chapters within the category of peer-review literature and news or magazine articles capturing activist accounts within the grey literature. We did not include blogs, society or social media pages, emails or tweets which may be considered a valuable further layer of grey literature (Adams et al., 2017) due to time constraints regarding retrievability and difficulty in determining credibility.

Peer-review literature was predominantly sourced via database searches. This was followed by a google search using the same terms applied to the database search to mine grey literature. Google has been used by other scholars as a viable way to mine further grey records beyond the databases (Bertels et al., 2010; Menezes & Kelliher, 2011). Both search types will now be discussed.

### Search Strategy

A first-round identification screening launched this review, identifying records between January 2010 and September 2020 because heightened interest in campus sexual violence stems from around 2010 in the UK in particular, but also from other countries such as the US (*Bovill et al., 2020). Other exclusion limiters were ‘English language’ and ‘higher education’ where databases permitted. Databases searched included British Education Index, British Humanities Index, Eric (EBSCO), Proquest, Scopus, Sociological Abstracts and Sage. Databases were divided between the two authors. Keywords used were sexual violence, sexual assault, and rape, which were combined in various ways with activism, university and university students. Other terms such as sexual harassment, cyber abuse, unwanted sexual contact, dating violence, domestic violence and bystander were used initially to establish robust terms, but were not garnering relevant results regarding activism, and so we did not continue to pursue these terms. Additionally, time and resources were a constraint on testing further terms. With the terms used in the search, repetition of sources was quickly established, and so, reasonable saturation of sources assumed to have been achieved.

This first round resulted in identification of (*n* = 825) potential returns. During this first-round, the authors met, for quality assurance purposes, to compare search strategies. This first round then consisted of assessing titles and skim reading abstracts or content to establish if records returned contained indicators for inclusion related to research questions. This narrowed returns from databases to (*n* = 126). A second-round identification screening of duplicates resulted in (*n* = 91) records.

This same strategy was then applied to a Google search, using the same terms above in various combinations but adapting strategy to this environment. Some authors advocate following up a consistent number of Google search returns, for example, the first few hundred presented in each search combination used (Bertels et al., 2010) and others, a consistent number of pages (Bowen et al., 2010). Eight Google pages were scanned for each combination of terms for relevant articles, whereupon a point of diminishing returns was established. Before selection, these were checked against the (*n* = 91) database records for replication. Therefore, a much smaller number of records were identified resulting in a further (*n* = 25) potential records, totalling (*n* = 116) records; initially stored in RefWorks.

**Figure 1.**
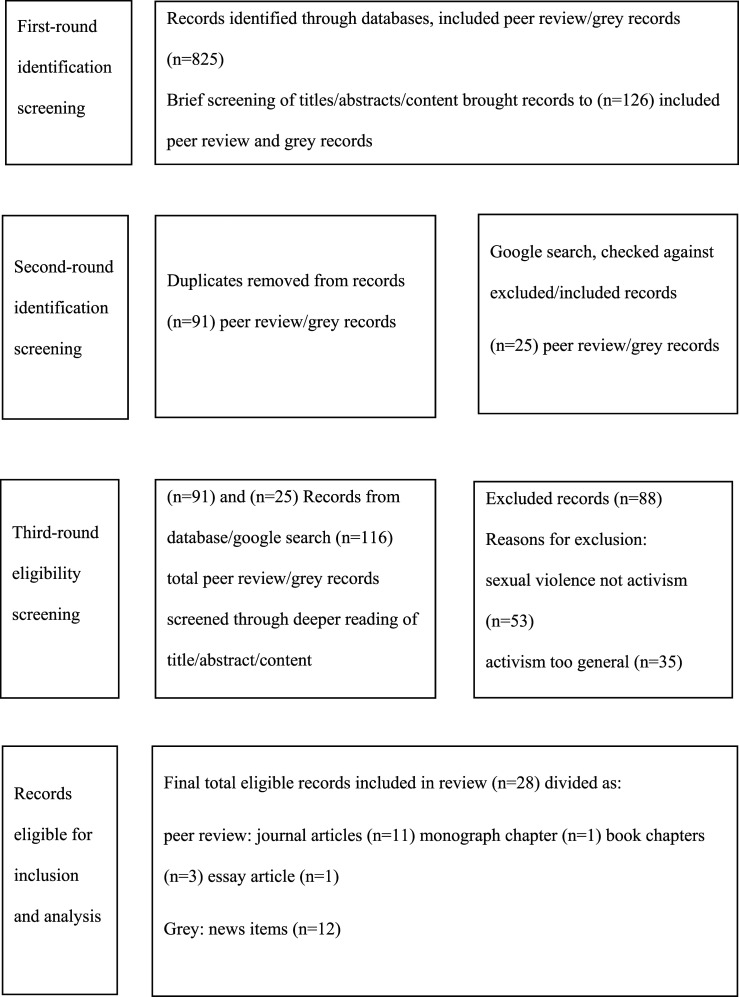
Search and Screening Results. Adapted from Fedina et al. (2018).

These (*n* = 116) records were then shared between the two authors of this study for a third-round eligibility screening during which each record was read in more depth for fit with the research questions. Those records established as fitting had reference lists scanned for any missed relevant records. Authors swapped records, checking 10% of each other’s included and excluded records for quality assurance purposes. This resulted in (*n* = 28) records for final inclusion and analysis scrutiny. See Figure 1 for search and screening results adapted from Fedina et al. (2018).

### Analysis

For analysis, peer review and grey literature were treated as separate units (Adams et al., 2017). Peters et al. (2015, p. 144) refer to this stage of the process in a scoping review as ‘charting the results’. This stage should present a logical summary of studies which we have displayed in two charting tables (Table 1 peer-review literature) (Table 2 grey literature). Each set of sources (both peer review and grey literature in Table 1 and Table 2) was assessed for the country or countries it originated from. A brief summary was given, denoting source type within Table 1 and Table 2., for example, journal, monograph and news article. The subject of each source of literature was also stated in this summary column. In the final vertical column of Table 1, we adopted the National Institute for Health and Care Guidance (NICE,2012, p. 72) critical appraisal checklist (CASP) for qualitative studies to assess the quality of the peer-review literature. This assessed the peer-review literature for how clear the purpose, context and focus to questions were, the relevance and range of studies referred to, validity and reliability of methods, rigour of analysis, strength of findings and recommendations, and if limitations were considered. An overall rating using CASP was determined using the below ratings (NICE, 2012, p. 216):

++ All or most of the checklist criteria have been fulfilled,
+ Some of the checklist criteria have been fulfilled,
– Few or no checklist criteria have been fulfilled

**Table 1.** Charting the results: peer-review literature.

Reference	Country	Summary	CASP^1^ (2017) overall rating ++, + or -
Bovill et al. (2020)	UK and US	Journal. Campus activism	+
Burman et al. (2020)	UK	Chapter. Activism coalition	+
Krause et al. (2017)	US	Journal. Activism advocates	+
Lewis & Marine (2018)	UK and US	Chapter. Student accounts	+
Lewis et al. (2018)	UK and US	Journal. Feminist resistance	++
Linder (2019a)	US	Journal. Theoretical activism	+
Linder (2019b)	US	Monograph chapter. Activist leadership	+
Linder & Myers (2017)	US	Journal. Campus activism	++
Linder et al. (2016)	US	Journal. Social media role	++
Linder et al. (2019a)	US	Journal. Minoritised experiences	++
Linder et al. (2019b)	US	Journal. Minoritised experiences	++
Marine & Lewis (2019)	UK and US	Journal. Gender equity activists	++
Marine & Trebasacci (2018)	US	Journal. Activist identity	++
Mitra (2015)	US	Essay article. ‘Carry that Weight’	_
Rentschler (2018)	US and Canada	Journal. Campus assault	_
Stenson (2020)	UK	Chapter. Sexual violence	+

^1^CASP. Critical Appraisal Skills Programme. A set of eight appraisal tools to be used as checklists when reading research.

**Table 2.** Charting the results: grey literature.

Reference	Country	Summary
Grigoriadis (2014)	US	Magazine article. Student accounts
Grinberg (2014)	US	News article. Student accounts
Karasek (2018)	US	News article. Student accounts
Kingkade (2014)	US	News article. Student accounts
Ludden (2014)	US	News article. Student accounts
Nathanson (2014)	US	Magazine article. Student accounts
Ortlip-sommers (2017)	US	News article. Student accounts
Smith (2014)	US	News article. Student accounts
Taylor (2020)	US	Digital media article. Student accounts
Vagianos (2019)	US	News article. Student accounts
Vella (2018)	US	News article. Student accounts
Whitford (2019)	US	News article. Student accounts

We determined an overall rating of ++ or + for all but two of the (*n* = 16) included peer-review articles. Thus, the transferability, trustworthiness and rigour of the studies were determined as robust. We included the two records which were rated as – on the CASP checklist, as we determined these records had important contributions to make in terms of ‘“why” activists “feel and behave the way they do’ (Campbell et al., 2020, p. 457, adapted from (Popay, 2005). Only the peer-review literature was assessed with CASP (2020), as the CASP checklist enables a review of transferability, trustworthiness and rigour of study findings when reading research (Campbell et al., 2020; Hannes & Macaitis, 2012). This kind of quality assurance is not expected to be found within the grey literature which is not research and so was not applied to Table 2.

Thematic analysis (Braun & Clarke, 2006) was adopted using NVivo12, importing the peer-review literature into one file, and the grey literature into another. Stage-one thematic analysis consisted of each author reading through their corpus of peer-review or grey literature. This enabled stage two where preliminary codes were constructed. Within this stage, we identified five initial broad themes under which we had identified 35 codes. These were recorded as nodes in NVivo12. In stage three, both authors moved text from the peer-review and grey literature as it applied under the nodes in NVivo12 using constant comparison between records and noting emerging concepts or concepts which had less analytic strength. In stage four, both authors met to discuss development of strongest themes and codes, and potential higher-order themes. Stage-five involved reviewing and naming themes, with data moved according to fit.

## Results

Research questions will be addressed under the themes of survival from harm, community, labour in the personal made public and power between activists and institutions. Table 3 sets out which sources helped to develop which theme and how each theme related to the research questions.

**Table 3.** Themes and relationship to the questions and the literature.

Theme	Research question	Peer-review literature	Grey literature
Survival from harm	1. What drives sexual violence survivors to become student sexual violence activists?	Bovill et al. (2020) Krause et al. (2020) Lewis & Marine (2018) Lewis et al. (2018) Linder (2019a) Linder (2019b) Linder & Myers (2017) Linder et al. (2016) Linder et al. (2019a)	Grigoriadis (2014) Grinberg (2014) Karasek (2018) Kingkade (2014) Ludden (2014) Nathanson (2014) Smith (2014) Taylor (2020) Vella (2018)
Community	2. What are the key positive and negative impacts for sexual violence survivors of their student sexual violence activism?	Lewis & Marine (2018) Lewis et al. (2018) Linder & Myers (2017) Linder et al. (2016) Marine & Lewis (2019) Marine & Trebisacci (2018)	Ludden (2014) Nathanson (2014) Ortlip-sommers (2017) Smith (2014) Taylor (2020) Vagianos (2019)
Labour in the personal made public	2. What are the key positive and negative impacts for sexual violence survivors of their student sexual violence activism?	Bovill et al. (2020) Krause et al. (2017) Lewis & Marine (2018) Linder et al. (2016) Linder et al. (2019a) Linder et al. (2019b) Marine & Trebasacci (2018) Mitra (2015)	Kingkade (2014) Ludden (2014) Nathanson (2014) Ortlip-sommers (2017) Taylor (2020) Vagianos (2019)
Power between activists and institutions	3. What does a greater understanding of survivors and their sexual violence activism tell us about response by higher education institutions?	Bovill et al. (2020) Burman et al. (2020) Krause et al. (2017) Lewis & Marine (2018) Lewis et al. (2018) Linder (2019a) Linder (2019b) Linder & Myers (2017) Linder et al. (2016) Linder et al. (2019a) Linder et al. (2019b) Rentschler (2018) Stenson (2020)	Grigoriadis (2014) Grinberg (2014) Karasek (2018) Karasek (2018) Ludden (2014) Nathanson (2014) Smith (2014) Vella (2018) Whitford (2019)

### Research Question One

#### What Drives Sexual Violence Survivors to Become Student Sexual Violence Activists?

##### Survival from Harm

Activism can be a way of surviving harm, both personal harm from experience of sexual violence and harm arising from the way institutions dealt with sexual violence complaints. Having experience of sexual violence is a common feature of student sexual violence activists (for example, *Linder & Myers, 2017; *Linder et al., 2016). All the activists identified in the grey literature had experienced sexual violence, as had most in the peer review, and saw activism as a way of dealing with these personal experiences (*Karasek, 2018; *Nathanson, 2014; *Taylor, 2020). One of the key drivers of activism for survivors was the harm that arises from the inadequate response from institutions towards sexual violence complaints. For example, feelings of betrayal and a desire to hold institutions to account resulting from poor handling of sexual violence complaints and disclosure, were prominent across the literature. The concept of ‘institutional betrayal’ was found across much of the peer-review literature (for example, *Lewis et al., 2018; *Linder, 2019a; *Linder, 2019b; *Linder & Myers, 2017; *Linder, et al., 2016). Poor complaint handling of the original incident of sexual violence by either the university or police was cited by many of the grey records as being the precipitating factor in sexual violence activism (for example, *Grigoriadis, 2014; *Grinberg, 2014; *Kingkade, 2014; *Ludden, 2014; *Nathanson, 2014; *Karasek, 2018; *Smith, 2014, *Vella, 2018). *Lewis and Marine (2018) note anger against poor handling and urgent concern regarding sexual violence, as a drive to activism. They also note that this extended beyond the activists’ immediate university environment to a sense of concern which ‘represents and reflects the state of gender relations and women’s lives’ more generally (*Lewis & Marine, 2018, p. 136).

Conversely, loyalty to an institution emerged as a further facet of survival from harm. Institutional loyalty emanated from a desire to encourage institutions to respond more effectively to complaints of sexual violence, alongside wanting to improve the institutional climate for future students (*Krause et al., 2017; *Linder & Myers, 2017, *Linder et al., 2019b). Activism can be a way to gain some sort of closure where institutional or legal recourse has not been effective; in this way, activists can be constructed as ‘agents of change’ (*Krause et al., 2017). Bovill & White, 2020 have also explored agentic change as an aspect of activism, noting that activists can be important in keeping the subject of sexual violence on the agenda within university environments that can be resistant or reluctant to acknowledge issues of sexual violence. Drawing on Kingdon’s (2010) work on ‘policy windows’, *Bovill et al., 2020 explore activists as ‘policy entrepreneurs’ who use these policy windows for change. Additionally, activists are key in keeping policy windows open long enough to instigate change, and there are ‘insiders’ and ‘outsiders’ who can influence the extent to which policy windows can be accessed by others (Kingdon, 2010). In answering question two and three below, we will further examine the support activists receive from ‘insiders’, such as administrations, institutions and administrative staff who can support or subvert the change activists are seeking to make.

### Research Question Two

#### What are the Key Positive and Negative Impacts for Sexual Violence Survivors of their Student Sexual Violence Activism?

##### Positive: Community

Through activism, survivors found community with other like-minded activists who had shared experiences of sexual violence. This extends the theme ‘survival from harm’ discussed previously. The literature demonstrates that within the relative safety of an activist community, activist consciousness of commonality of experience can emerge, new ideas can flourish and agency can be activated. *Marine and Trebisacci (2018) note how shared grievances and a collective purpose are essential in building activist identity. *Lewis et al. (2018) note that establishing community was ‘reassuring’, ‘affirming’ and ‘empowering’ for many women and could help raise a collective consciousness of gender issues and accountability. A lack of accountability and recourse has been termed a second assault at the hands of institutions mishandling disclosures (*Linder & Myers, 2017) and activist communities can be a resource to deal with the aftermath of this. *Lewis and Marine (2018) *Linder et al. (2016) and *Marine and Lewis (2019) show that activists can find safety in communities of activism, that can build confidence, reflection and the ability to argue for what you believe in, enabling activists to speak out and speak up to dominant ideology. *Lewis and Marine (2018, p. 130) argue that activism is important to ‘create cultures which support freedom, resistance and respect’ so that normalisation of sexual violence is challenged, and perpetrators and institutions become accountable.

Friendship through activism (*Lewis & Marine, 2018; *Lewis et al., 2018; *Marine & Lewis, 2019) was cited by participants as a positive outcome of activism which further cemented community and collective consciousness of the experience of sexual violence. The common experience of taking part in marches, protests and other forms of activism helps to develop friendships and form bonds. Solidarity between survivors who may feel isolated was also identified as a positive impact of activism in the grey literature (*Ludden, 2014; *Nathanson, 2014; *Ortlip-Sommers, 2017; *Smith, 2014; *Taylor, 2020). The grey literature further supports the power of activist communities, finding that activism can be healing, bringing a sense of empowerment (*Vagianos, 2019). This empowerment can take multiple forms, the power to help other sexual violence survivors, a sense of self-empowerment and empowerment to encourage sexual violence activism in others (*Taylor, 2020).

#### Negative: labour in the personal made public.

Sexual violence activism is hard laborious work which, coupled with deeply personal experiences of sexual violence often being made public, can have a high detriment to personal and public well-being for survivors. The role of activism to enable survivors to ‘witness, name and challenge’ sexual violence is noted by *Lewis and Marine (2018, p. 130) as an important mechanism of activism. Particularly prevalent within the grey literature was the importance of combating the isolation of survivors by bringing into the public sphere, trauma that is often experienced alone (*Nathanson, 2014; *Ortlip-Sommers, 2017; *Taylor, 2020). However, with this can come too much negative attention which can be hard to deal with (*Nathanson, 2014).

Emotional, physical, mental and social labour resulting from sexual violence activism was highlighted throughout the literature, together with the public nature of having your story known (for example, *Bovill et al., 2020; *Krause et al., 2017; *Linder et al., 2016; *Linder et al., 2019a; *Linder et al., 2019b; *Marine & Trebisacci, 2018; *Mitra, 2015). Accounts of backlash, discrimination and isolation, poor academic performance, burnout, isolation from the university experience and a sense of futility emerged.

*Linder et al. (2019b) note that whilst engagement in activism can lead to growth, it is a form of unpaid labour, described by *Marine and Trebisacci (2018) as invisible labour, which can take students away from study. In question one (previously), *Bovill et al., 2020 develop Kingdon’s (2010) ideas of ‘insiders’ and ‘outsiders’, noting that amongst ‘outsiders’, special interest groups emerge and this can include student activists. These special interest groups can have the opportunity to ‘form bridges’ with those on the ‘inside’, which might include institutions and administrators. However, special interest groups are likely to have greater impact if they organise in particular ways. This includes persistence and a sustained approach which takes an investment of time, energy and resources, all of which require significant labour. The grey literature bears this picture of labour out, further noting activism was found to be difficult and dangerous, leading to ridicule, threat and attacks (*Kingkade, 2014) as activists were put into the spotlight (*Ludden, 2014). This is particularly the case on small campuses, where everyone knows everyone else (*Vagianos, 2019). The labour required to conduct activism has the potential for re-traumatisation and triggering to occur (*Ortlip-Sommers, 2017).

*Mitra (2015) further explores the labour of sexual violence activism when reviewing the collective action arising from ‘Carry that Weight’, a performance piece by Emma Sulkowicz, a sexual violence survivor. Sulkowicz carried their dorm mattress around campus to signify the weight they carried after their rape and to protest for their perpetrator’s removal from campus. This piece of performance art captured popular imagination and eight weeks after it began, 130 universities across five countries were involved in ‘collective carries’ in solidarity with survivors. *Mitra (2015) continues their analysis of ‘Carry that Weight’, noting the mattress signified a metaphorical burden, the burden of the assault and the burden of proof that falls on the survivor. Sulkowicz, when interviewed in *Nathanson (2014), stated that they used their ‘privileged position’ to bring attention to the issue of sexual violence but in doing so explained that consequences to personal life can occur. For example, highly successful protests possibly lead to too much publicity for the sexual violence activists (*Nathanson, 2014), and this sometimes resulted in backlash.

### Research Question Three

#### What does a Greater Understanding of Survivors and Their Sexual Violence Activism Tell us About Response by Higher Education Institutions?

##### Power Between Activists and Institutions

To act within a power-conscious framework involves ongoing reflection upon and awareness of the dynamics inherent in power, privilege and oppression, and pushes people and institutions ‘to consider the various way power and privilege influence…dominant group members’ investment in and benefit from a system of domination’ (*Linder, 2019a, p. 23). The literature surveyed shows clearly that one of the key drivers of student sexual violence activism is the inadequate response from universities and colleges towards sexual violence complaints. In this section, and the discussion below, the identified theme of the power imbalance between student sexual violence activists and institutions will be examined, showing that institutions are able to use their power to dismiss and neutralise actions of the activists. These sections will also show that activists can and do resist this through activism. This literature review seeks to make the case that rather than institutions and activists being almost adversarial, sexual violence activists and their activism can be a resource for institutions to work with rather than against in handling issues of sexual violence on campus.

Sexual violence activists face unique challenges, as their universities and colleges may fear institutional harm or damage to their reputation (*Lewis et al., 2018; *Linder, 2019a; *Linder, 2019b; *Linder & Myers, 2017; *Linder et al., 2016; *Linder et al., 2019b). A high presence of activism against sexual violence on campus may be viewed as a sign of high levels of sexual violence. Such activism can be seen as underscoring poor institutional behaviour and practice and a need for institutional change. To understand the challenges faced by students taking part in sexual violence activism, it is useful to contrast types of activism. Some types of activism are viewed by institutions as more positive than others. For example, protesting for environmental causes. Such activism is unlikely to have a direct impact on the reputation of the institution and may therefore be seen by the institution as a praiseworthy form of citizenship or leadership development to be encouraged (*Linder, 2019a). Indeed, if a university is seen as environmentally aware and actively instigating positive change it can be viewed as an aspect worth highlighting. In contrast, sexual violence activism is more often viewed by institutions as a less desirable form of activism which can be uncomfortable for institutions (*Whitford, 2019). Particularly when, as has been shown previously, it arises from accusations of poor handling of sexual violence complaints by these institutions (see, for example, *Grigoriadis, 2014; *Grinberg, 2014; *Karasek, 2018; *Kingkade, 2014; *Ludden, 2014; *Nathanson, 2014; *Rentschler, 2018; *Smith, 2014).

In order to avoid reputational harm, the literature review shows that institutions can use their power to react to sexual violence activism in different ways by ignoring the complaint, mishandling the complaint and waiting out the problem. Institutions can use their power strategically, by hiding the extent of sexual violence on their campus or by making administrative procedures deliberately obtuse so that it is difficult to complain or to seek action from institutions. This literature review shows that institutions may publicly decry sexual violence, whilst not actually confronting instances of sexual violence within their own institutions, or even meeting with student sexual violence survivors, complainants or activists (*Linder, 2019a). Activists can instrumentally fight back against this, but it is rarer, and institutions may be banking on this rarity, whereas a more effective approach could be to work with the activists to bring about resolution. However, as documented in the many survivor stories in *Grigoriadis (2014), institutions are often more concerned with mitigation which protects reputation, rather than actions which are led by a need to address the situations of sexual violence on campus. Institutions which ignore sexual violence have been described as causing more trauma to those who experience sexual violence through ‘institutional betrayal’ (*Linder & Myers, 2017) or through attempts to de-legitimise student claims by constructing students as reactive and overly sensitive (Rentschler, 2018). Such action may highlight limited institutional commitment to change (*Krause et al., 2017; *Lewis & Marine, 2018; *Linder & Myers, 2017; *Linder et al., 2019a; *Linder et al., 2019b).

As discussed previously, there is significant evidence in the grey literature that complaints of sexual violence are mishandled not only by universities and colleges, but also by external agencies like the police (see, for example, *Grigoriadis, 2014; *Grinberg, 2014; *Kingkade, 2014; *Ludden, 2014; *Nathanson, 2014; *Karasek, 2018; *Smith, 2014, *Vella, 2018, *Lewis & Marine, 2018). *Grinberg (2014) notes that the level of resistance to campus cultural change is demonstrated by sexual violence survivor and activist accounts of institutions stalling complaint procedures and being slow in providing information requested by survivors or activists. For example, when student Joanna Espinosa filed a complaint against her boyfriend, she said that the university reporting process was so traumatising that she made a federal complaint against the university (*Grinberg, 2014). *Grigoriadis (2014) notes of Emma Sulkowicz’s complaint to university administrators, they made errors in her notes which were incomplete and inaccurate. Annie Clark and Andrea Pino also put the spotlight on their institutional response. Both accused their US universities of a lax, approach to their disclosures of rape.

However, power imbalances can be disrupted, and an example of this is demonstrated through Pino and Clark’s nationwide campaign where they co-founded ‘End Rape on Campus’ (EROC, 2013). They went on to work with a large network of activists educating survivors of their rights regarding disclosures of sexual violence and helping them to file complaints against institutions through Title IX^3^ legislation to force universities in the US to address sexual violence disclosures. At the time of *Grigoriadis’ (2014, p. 6) article there were 78 US colleges under investigation. Another example of power exchange through activism can be found in the campaigning of Sulkowicz (‘Carry that Weight’ campaign). This protest went national and global with mattress carries across campuses and pointed ‘a finger not only at assailants but also…the ivory tower of privilege’ (*Grigoriadis, 2014, p. 2).

A final way in which the literature surveyed showed that institutions were able to use their power to neutralise the actions of sexual violence activists is by ‘waiting out’ the problem (*Bovill et al., 2020; *Lewis & Marine, 2018). The research shows that many forms of sexual violence activism are short-lived, campaigns may have limited resources and institutions may be instrumental in restricting access to resources and making processes and access to expertise complex. This issue is compounded by the regular change in the student cohort and the temporality of students (*Stenson, 2020, p. 114) can be a powerful tool that institutions use to stall or detract from sexual violence activism. *Ludden (2014, p. 4) states that institutions are ‘morally reprehensible’ in the ways in which the burden for change falls to students and notes that students graduate and ‘College administrators know this,"...They know that if they can just hunker down and weather a crisis, that group of students is going to graduate sooner or later’. However, *Ludden (2014, p. 4) points to the importance of alumni, stating ‘but alumni are always around. We have a lot of influence…we still have a lot of say on what kind of culture we have on our campuses’. *Stenson (2020) refers to institutional impermanence or a lack of commitment to change, and notes both the benefits of activists and institutions working together and the challenges, whereby institutionalisation can lead to de-politicisation of issues which can then result in a loss of momentum. However, ultimately success of student activist campaigns hinges on institutional support. *Stenson (2020) notes that activists should be at the forefront of change where they are listened to, resourced and supported, but institutions must continue to carry this on when one set of activists leave. Long-standing change is achieved over time by new student activists continuing the work of previous student activists, and institutional support is essential for this.

The research surveyed shows that social media can play an important role in keeping sexual violence activism alive from one generation of student activists to the next. *Linder et al. (2016) note that social media activism can help shape a collective identity across time and space, and *Rentschler (2018) highlights this capacity too, noting media use can make activism more visible and therefore harder to erase and online activist networking enables scalability of activism across universities. *Bovill et al. (2020) also consider the capacity of online student sexual violence activism to create a place where previous cohorts of student sexual violence activists can record their actions forming “institutional memory” (*Whitford, 2019). The rhythm of university life, which includes long holidays, periods of extreme workload and the passing of one student cohort to the next, can be the greatest barrier to activism. Some institutions may use this rhythm to their advantage to draw attention away from issues of sexual violence by waiting for interest to wane or student activists to leave. Therefore, *Whitford (2019) notes that it is crucial to create an online space where previous information can live so that each student cohort can carry on the work of previous cohorts. Whilst noting the importance of social media in the continuation of activism, *Bovill et al. (2020) suggest that social media records can only offer a partial solution to the constant issue of student turnover, which institutions can benefit from in terms of power play and keeping the issue of sexual violence under the radar. The following discussion will examine what else can support continuation of change from sexual violence activism over the longer term.

## Discussion

In March (2021), following the movement set off by Sarah Everard’s^4^ death in the UK, a UK university campus was occupied by campus sexual violence activists who said ‘they won’t back down until the university implements their demands, which include improving student wellbeing services, disciplinary procedures and sexual violence education for both staff and students’ (Oakes, 2021, p. 2). The article continues, stating that while the problem of sexual violence on campus is widespread, ‘universities have a history of failing to react sufficiently to reports of sexual violence on campus’ (Oakes, 2021, p. 2). Whilst resolutions have not necessarily occurred from this action, the university has stated its openness to ‘listening to demands’, and though this movement is from a UK vantage point, it is relevant in a much broader context as it represents an example of a ‘policy window’ (Kingdon, 2010). Referring back to *Bovill et al. (2020), this movement, precipitated by Sarah Everard’s murder, and other movements like it demonstrate the potential for activism and activists acting as ‘policy entrepreneurs’ (Kingdon, 2010) to grasp these moments to bring about change. However, working in community with proactive supportive institutions on board is key.

### Implications for Practice, Policy and Research

The four themes identified in the review – survival from harm, community, labour in the personal made public and power between activists and institutions – suggest ways in which activists, institutions and researchers might work together to address questions of sexual violence on campus and the impact of activism on sexual violence survivors.

### Practice and Policy

#### Create Community

The importance for sexual violence activists of being part of a community was a key finding in the review. Lewis and Marine (2020, p. 1) note that ‘activism on campuses has been particularly fruitful when students and faculty coalesce in opposition to sexual harassment’. They suggest that this kind of collaborative community can lead to policy and practice changes which are meaningful, citing a ‘whole university approach’. They give an example from a Scottish university developed by *Burman et al. (2020) which put together a coalition of activists, academics, faculty staff and external agencies and joined forces with a neighbouring university. This resulted in a grassroots movement emerging, which developed a manifesto with seven demands including training for staff and students to be mandated, improvement to support services for students and making sexual violence an explicit breach of student conduct. There was apprehension from senior university members related to concerns of institutional harm or ‘reputational risk posed by focusing on this issue’ (*Burman et al., 2020, p. 175). However, working together, staff and students achieved changes to the Code of Student Conduct, improved support information for survivors, developed peer-led workshops and created a toolkit for university staff training. This approach demonstrates that lobbying through strong collective action and alliances are crucial to persuade institutions to commit to real cultural change and provide resources to do this (*Burman et al., 2020; Donovan et al., 2020). Such an approach indicates that a mutually beneficial outcome can be achieved through sexual violence activists promoting their universities as being proactive in their approach, rather than reactive.

#### See Activism as Work

A sub-theme identified in the literature was that sexual violence activism can become a form of unpaid, invisible labour, which takes students away from their studies (*Marine & Trebisacci, 2018). This could be addressed both by potentially remunerating activists for their activism (*Linder, 2019b) and by promoting sexual violence activism amongst all students on campus. One way to address this is to encourage students who have not experienced sexual violence to participate in sexual violence activism. There is encouraging evidence from training programmes for students who have not experienced sexual violence that ‘those who participate in programs or courses which discuss the arena of sexual violence are more likely to intervene and less likely to be ignorant to’ sexual violence behaviours (Bovill & White, 2020, p. 6). Such programmes could take some pressure off students who have themselves experienced sexual violence.

The UN Women is an organisation which collaborates with the legislature and United Nation’s organisations to tackle gender inequality, and they have produced guidance notes for universities, university administrators, students and other stakeholders. These are based on a human rights approach, which is survivor-centred and includes perpetrator accountability (UN Women, 2018). The notes include a list of 10 actions, which encompass putting in place protocols outlining procedures for claims of sexual violence and recognising the importance of activism and activists as agents of change. Action 9 ‘awareness raising and by-stander programmes’ looks at a range of initiatives which spreads the work of changing cultures across a wider audience. ‘Green Dot’, ‘Bringing in the Bystander’ and ‘Stop Sexual Violence’ are all programmes emanating from the US and ‘The Intervention Initiative’ from the UK. The programmes all train community members to recognise and respond to potentially harmful situations ‘to shift social norms so as to prevent violence from occurring’ and ‘raise awareness by educating students about the role each individual must play in creating a more positive, empowering campus environment’ (UN Women, 2018, p. 28). This guidance also explicitly states recommendations for students and staff to work together as experts ‘on gender-related issues, VAW, or student safety, to support dissemination of messages and student-led activism’ (UN Women, 2018, p. 13). Additionally, they note ‘it is important for faculty and staff to be effective allies of students by offering advice and other forms of support to student-led activism for institutional change’ (UN Women, 2018, p. 28).

Activism is often separated from academic leadership in universities, and *Linder (2019b) recommends institutions should formally support activism through the application of resources and sharing of expertise. Such coalitions would enable activists to navigate complex institutional systems, engage in long-term planning and identify and achieve desired outcomes in collaborative, rather than adversarial, ways. Instead of administrative processes being hidden from view, university leadership should work to make them transparent and accessible to understand, support and critique. This literature review has also identified that a reason why achieving lasting change is inherently difficult is because the student cohort was inevitably constantly changing. It is recommended that institutions work with sexual violence activists to document their activism so that the successes and lessons learned are passed from one cohort of activists to the next, rather than have progress fade from view.

#### Celebrate Activism, Rather than Fearing Reputational Damage

The literature review has shown that one of the key drivers of sexual violence activism is the poor response by institutions. Activist intent can be to hold institutions to account for harmful behaviours in a very public manner. Institutions put up barriers to this process, fearing institutional harm and reputational risk, particularly the impact that this might have on student recruitment if their campus were seen to have a problem with sexual violence. This paper has shown that it is highly likely that all universities and colleges have a problem with sexual violence on campus, thus institutions need awareness of (1) the potential futility of attempting to hide the problem and (2) alternative ways of looking at the issue. One way of reframing the issue could be that rather than hiding the problem of sexual violence on campus, the institution’s reputation could be based on the fact that it is actively addressing sexual violence. Donovan et al. (2020) who conducted survey research and interviews with academics on gender-based violence, noted from one of the academics participating in the research that:

It should be possible to put the alternative argument that an institution’s reputation is built on the fact that it undertakes this work, thereby flagging to the rest of society that it takes the issue seriously…this argument is difficult to make, and in any case “we should be able to say ‘this is what you should be doing’ irrespective of whether it brings students in or not” (Donovan et al., 2020, p. 136).

Furthermore, institutions must acknowledge that in trying to ignore disclosures or obstruct investigations, the issue of sexual violence does not disappear. Institutional inactivity, denial or poor response to sexual violence on campus can be futile, as the institution does not (always) remain unharmed. Activist response to institutional failures in the handling of instances of sexual violence on campus has led to the amplification of the issue and has forced changes to occur, despite institutional obstruction (Witt, 2015). Thus, in attempting to avoid reputational damage, institutions may highlight institutional shortcomings instead.

### Research

There is a need for research that illuminates the experiences of student activists who are survivors of sexual violence research. Research in similar fields suggests that the most fruitful research involves power-sharing between researchers and participants and as such is collaborative and conducted in coalition with activists throughout the process (see, for example, Ragavan et al., 2020; Ghanbarpour et al., 2018). In this way, new knowledge that is generated and any resulting policy change is informed by robust evidence, based on experience. In the US, *Krause et al. (2017, p. 216) explored research with Campus Peer Advocates, which is a student-led peer advocacy program providing training ‘about sexual consent, gender inequitable relational power, institutional policy and support services for survivors of sexual assault’. They make strong recommendations that researchers work with activists to foster lasting change. The recommendations they make stem from a feminist praxis. This includes genuine collaboration which challenges discourse and cultures, and comes from an epistemological standpoint which assumes the lived experience proffers a powerful position of truth and knowledge which transfers power to the oppressed.

### Limitations

Though this literature review was methodically followed through recommended steps (Adams et al., 2017; Peters et al., 2015), records may have been missed or excluded. Balancing breadth and depth, time constraints, publishing word limits and exclusion of studies published in languages other than English are all potential constraints of this study noted in other scoping reviews (Pham et al., 2014). Additionally, included sources predominantly represent the UK and US perspectives as currently, this dominates research in this area. This is a significant limitation as most research regarding sexual violence and higher education is noted to be taken from a Western context; therefore, this does not necessarily represent the global experiences of students (Gómez, 2019; Zounlome et al., 2019). Additionally, the studies included in the review focussed (almost) exclusively on women students and male-perpetrated sexual violence, reflecting current research findings, with rates of reported sexual violence towards men lower (Borumandnia et al., 2020) and barriers to male reporting of sexual violence, such as stigma (Hammond & Ioannou, 2016). Therefore, findings are not necessarily transferable to some populations, such as male students who have experienced sexual violence. Choosing to focus on direct experience of sexual violence was a pragmatic choice of the authors due to time and word count constraint of this particular paper. However, we note that this excludes the valuable position of ally activism and its power in changing the sexual violence landscape which could form the basis of a separate paper in its own right.

## Conclusion

This literature review is significant as it highlights a recently emerging field within one body of work, aligning with and extending the findings of the leaders in the field of sexual violence research and activism. Findings are novel in that they highlight the voice of student sexual violence activists, not only within the peer-review literature but also within the grey literature and make a strong case for formalising coalition building between activists and institutions.

This review established that poor institutional or inadequate legal response drives student sexual violence activism. It also found that student sexual violence activism was often operated as a tactic of survival from harm, to hold institutions to account for poor handling of complaints and a desire to improve the sexual violence landscape for future generations. This review demonstrated that student sexual violence activism has the capacity to build community with like-minded others who share the experience of sexual violence. Finally, this review helped to determine potential ways forward to work with student sexual violence activists so that lasting cultural change, that is aware of power imbalances and their impacts, can start to occur in ways that are beneficial for the whole community. The literature has pointed to institutions as potential sources of harm, but it also points to institutions as places where good work to challenge sexual violence on campus can and does occur. Institutions working in coalition with, instead of division from, student sexual violence survivors who become sexual violence activists have the potential to lead to authentic and lasting cultural change.

## Notes

1. * Next to a reference denotes a reference which has been included as part of this literature review.

2. The findings from Revolt Sexual Assault include both sexual harassment and sexual assault. The 70% figure would include areas such as verbal harassment and sexual commenting.

3. Title IX is a federal civil rights law in the US resulting from Education Amendments in 1972 which states that ‘no person in the United States shall, on the basis of sex, be excluded from participation in, be denied the benefits of, or be subjected to discrimination under any education program or activity receiving Federal financial assistance’ (U.S Department of Education, 2021, p. 1). All US institutions in receipt of federal funding must have a designated Title IX co-ordinator.

4. 3 March 2021, Sarah Everard was kidnapped in London by Metropolitan Police Officer Wayne Couzens, who falsely arrested her under the pretence of having breached COVID-19 regulations. Couzens raped and strangled Sarah.
